# Smartphone-based markers of social connectivity in schizophrenia and bipolar disorder

**DOI:** 10.1038/s44277-024-00013-w

**Published:** 2024-07-24

**Authors:** Linda Valeri, Xiaoxuan Cai, Habiballah Rahimi Eichi, Einat Liebenthal, Scott L. Rauch, Dost Ongur, Russell Schutt, Lisa Dixon, Jukka-Pekka Onnela, Justin Baker

**Affiliations:** 1 Department of Biostatistics, Columbia University Mailman School of Public Health, New York, NY USA; 2 Department of Epidemiology, Harvard T.H. Chan School of Public Health, Boston, MA USA; 3 Department of Statistics, Ohio State University, Columbus, OH USA; 4 Department of Psychiatry, Harvard Medical School, Boston, MA USA; 5 McLean Hospital, Belmont, MA USA; 6 Department of Psychiatry, Harvard Medical School and Beth Israel Deaconess Medical Center, Boston, MA USA; 7 Department of Psychiatry, Columbia University Medical Center, New York, NY USA; 8 Department of Biostatistics, Harvard T.H. Chan School of Public Health, Boston, MA USA

**Keywords:** Predictive markers, Psychiatric disorders

## Abstract

Social isolation and social impairment are hallmarks of progression as well as predictors of relapse in psychiatric disorders. We conducted a pilot study to assess the feasibility of sensing the social activity phenotype and loneliness using active and passive markers collected using a smartphone application. The study included 9 schizophrenia and bipolar disorder patients followed in the Bipolar Longitudinal study for at least 1 month and for whom mobile communication data was collected using the Beiwe smartphone application. Subjects completed daily surveys on digital and in-person social activity, and feelings of being outgoing or lonely. We described the level and variability of social activity features. We employed k-means clustering to identify “important contacts”. Further, we investigated whether social network-derived features of mobile communication are independent predictors of weekly counts of outgoing calls and text, weekly average self-reported digital social activity, and loneliness using mixed effect models and clustering with dynamic time warping distance. Subjects were followed between 5 and 208 weeks (number of days of observation = 2538). The k-means cluster analysis approach identified the number of “important contacts” among close friends and family members as reported in clinical interviews. The cluster analysis and longitudinal regression analysis indicate that the number of individuals a person communicates with on their phone is an independent predictor of perceived loneliness, with stronger evidence when “important contacts” only are included. This study provides preliminary evidence that the number of “important contacts” a person communicates with on their phone is a promising marker to capture subjects’ engagement in mobile communication activity and perceived loneliness.

## Lay summary

The work of Valeri et al. gives insights into the value of adopting of a social network perspective for digital phenotyping of social activity and loneliness using call and text data in SMI populations. Mobile communication network degree of “important contacts“ inferred using a clustering approach is an independent predictor of self-reported loneliness and social activity.

## Introduction

Growing evidence supports the feasibility of utilizing mobile technologies to measure activity related to mental illness in patients with bipolar disorder (BD) and schizophrenia (SZ). Social isolation and loneliness can heighten the risk of exacerbating psychotic and depressive symptoms, leading to relapse. By leveraging smartphones, behavioral markers of mental illness can be derived to complement biological markers, self-reports in the clinic, and potentially identify behavioral treatment targets. Real-time information on social activity can provide additional insights to patients’ lived experiences and inform timely interventions. To fully realize the potential of digital phenotyping for social behavior in severe mental illness, robust research is needed to interpret digital traces of social behavior in relation to patients’ perceptions, life events, and clinical status.

Research on digital phenotyping of social activity in relation to mental illness and loneliness is rapidly advancing, with promising findings. Studies have demonstrated that passive sensing of behavioral “stability” in mobile social activity and sleep can predict symptoms and relapses in SZ [1–3]. For instance, reductions in outgoing phone calls and text messages have been identified as significant predictors of relapses in SZ [4]. Additionally, mobility and speech activity have been found to predict loneliness in SZ patients [5]. Mobile communication via call and text is a common form of social interaction entertained by individuals living with severe mental illness [4] and a recently published review [6] indicated that frequency and lengths of calls are commonly used passive markers of social activity in digital Psychiatry studies. However, despite the emerging evidence, current studies have certain limitations. Many studies do not integrate mobile traces of social activity with self-report and lack a comprehensive social theoretical framework for formal testing. Short follow-up durations also limit the impact of findings. The feasibility of collecting real-time information on social activity in individuals with BD disorder and SZ remains unclear. Furthermore, it is important to determine whether adopting a social network perspective in processing call and text data, rather than using more common features, such as frequency and length of activity, can enhance the characterization of perceived social activity and loneliness in these populations.

Integrating smartphone-derived traces of social activity with self-reports of in-person and digital social interactions is crucial but requires establishing best practices. Digital phenotyping holds great promise in capturing real-time social behavior to provide information that could enable timelier therapeutic (or even preventative) intervention in psychiatry. To fully harness this potential, it is crucial to validate phone-based features while anchoring the validation process in social theories pertaining to mental illness. Loneliness, with its significant impact on mental and physical health, has emerged as a critical factor to consider [7]. In the SZ patient population, loneliness has been shown to worsen negative symptoms and impair social functioning [8]. Similarly, in patients with BD illness, loneliness has been correlated with depressive symptoms [7]. Individuals with severe mental illness have been found to benefit from frequent contact with close friends with reported increases in adherence to treatment [9] and functional outcomes [10]. Information about social activity involving close friends is hard to obtain on a consistent basis but could be derived from passive data. No study thus far has attempted to identify important contacts from call and text logs in digital studies of SZ/BD nor has evaluated communication network features involving important contacts as predictors of loneliness of SZ/BD. Considering other health domains, in a recent study of SZ/BD patients, our group found that daily medicine ingestions, which were ascertained through a biosensor, were significantly associated with two communication metrics: the number of individuals whom the participants called (known as their social/communication network degree) and the duration of outgoing calls [11]. Acknowledging the significance of social activity, our study aims to assess the relationship between smartphone-derived measures of digital social interaction with self-reported measures of digital social interaction and in-person social activity. By doing so, we aim to enhance the measurement of actionable behavioral targets related to social activity. Additionally, we recognize the potential role of loneliness as a mediator for interventions that promote social activity and improve patients’ quality of life. Therefore, we also aim to investigate the association between mobile communication features and loneliness, providing additional validation for this construct [8]. Through these investigations, we aim to contribute to the understanding of how smartphones can inform interventions that enhance social activity, alleviate loneliness, and ultimately improve the quality of life for individuals with psychiatric disorders.

To accomplish this purpose, we conducted a pilot study to validate digital traces of social behavior and identify the key features that best describe patients’ engagement in social activity, which in turn are associated with the perception of loneliness. Our team has established a multi-year cohort study for digital phenotyping that followed 74 severely mentally ill patients. The cohort study was designed to follow for an extended period (the study is also referred to as the ‘one hundred person-year study’) a transdiagnostics sample of individuals who experienced psychosis to capture variability and trends in symptoms and behaviors in the natural environment. The goal of the Bipolar Longitudinal Study was to examine the naturalistic course of illness in a cohort of individuals with severe mental illness who experience clinically significant fluctuations in their mood and/or cognition. Because individuals with BD and SZ often share both genetic liability as well as many syndromic features, our transdiagnostic approach was intended to expose both shared and unique features of individuals experiencing either a primary psychotic condition or a primary affective condition, including the construct of social withdrawal. Moreover, by utilizing an intensive longitudinal assessment design, we sought to establish the extent to which individual patterns may be more predictive than patterns derived from the diagnosis category. This study was carried out in a subset of 9 patients (number of observations = 2538) whose use of an Android phone made it possible for us to extract passive data on mobile communication. In this feasibility study we did not want to exclude participants further and adopted an n-of-1 paradigm of study to accommodate patient heterogeneity in follow-up time and diagnosis [12].

The overarching goal of the study is to evaluate the use of participant communication network features, which have been developed in the field of network science, to quantify the correlation of actual call and text activity (observed without error from the communication logs of the phone) with self-reported social activity and loneliness. To achieve this goal, we first show the feasibility of extracting and filtering mobile communication logs collected across multiple years that may capture patients’ immediate social network, identify contacts that are in close relationship with the participants, denoted here “important contacts”, and integrate these passive data streams with daily survey data. To preserve participant privacy, note that the phone numbers of their contacts collected as part of communication logs were hashed on the phone prior to data uploading, i.e., contacts remained anonymous to the investigators. Second, we provide preliminary evidence of whether social network features of mobile communication are independent predictors of patients’ engagement in mobile communication (captured by outgoing counts of calls and text), patients perceived digital social activity and perceived extent of loneliness. Third, we test whether the identification of “important contacts” further improves our ability to capture patient-initiated mobile activity, patients’ digital social activity, and loneliness perceptions using social network features that capture mobile network size (degree) and interactivity of mobile communication (reciprocity). We hypothesize that incorporating social network features of mobile communication can improve our characterization of patient’s engagement in social activity and perceptions of loneliness in SZ and BD disorder, providing preliminary, fine-grained, evidence of social networks, passively monitored without requiring any patients’ reporting effort, being a potential target for treatment of severe mental illnesses.

## Materials and methods

### Overview of study design and study population

A total of 74 individuals diagnosed with severe mental illness were enrolled in the Bipolar Longitudinal study between 2015 and 2021 and followed for up to 4 years. Individuals diagnosed with severe affective and psychotic disorders were eligible to enroll in the study. This research received approval from the Mass General Brigham Institutional Review Board, and prior to their participation, participants provided informed consent. Individuals responded to a daily 31-item mobile survey questionnaire. Patients participated in screening, baseline, and monthly in-clinic visits throughout the follow-up that spanned from 1 week to 4 years. Mobile communication activity was available for 10 patients enrolled between 2015 and 2019. A subset of 9 patients were followed for at least one month.

### Smartphone data collection

Participants installed the Beiwe smartphone application on their personal smartphones. Beiwe is an open-source, end-to-end encrypted digital phenotyping platform that consists of Android and iOS smartphone applications, a web-based platform for study setup, HIPAA-compliant cloud-based data storage, and a data analysis back-end [13]. The smartphone application was configured to send daily reminders at 5 pm ET (eastern time) to fill a 31-item survey and to collect without any effort from the patient mobile call and text logs. All data were collected and stored in compliance with local, state, and national laws, and all regulations and policies.

### Measures

At baseline demographic information (age, gender, race) and patient’s diagnosis were recorded. The daily 31-item survey included questions on mood, psychotic symptoms, alcohol and coffee consumption, social behavior, physical activity, and sleep. The social activity items included questions on in-person and digital social activity (how much active in in-person social activity have you been? 1 = almost no interaction, 2 = little interaction more than superficial, 3 = meaningful interaction, 4 = an unusually deep interaction; how much active in digital social activity have you been? 1 = almost no interaction, 2 = little interaction more than superficial, 3 = meaningful interaction, 4 = an unusually deep interaction), the extent of feeling outgoing, and extent of feeling lonely (how outgoing do you feel?, 1 = not at all, 2 = a little, 3 = moderately, 4 = extremely; how lonely do you feel?, 1 = not at all, 2 = a little, 3 = moderately, 4 = extremely). We extracted anonymized phone communication logs and summarized them using the following weekly features: count of incoming and outgoing calls and texts, and duration of incoming and outgoing calls. We also extracted two measures of social activity motivated by the social network literature. The first feature is the number of unique contacts via call and text in each week, capturing mobile communication social network degree. The second feature is the count of incoming and outgoing calls and text exchanges with each contact within a week. This latter measure, which we call reciprocity, is an indicator of whether the patient is involved in sustained and interactive mobile communication with their contacts. Primary outcomes of our study include the count of outgoing calls and texts, digital social activity, and loneliness self-report. Primary predictors include reciprocity of calls and texts and a number of unique people the patient is in contact with via calls and texts. Having a close, mutually confiding relationship is critical in reducing psychological symptoms [14]. Therefore, “important contacts” also referred to in this article as “key contacts” are also identified for each participant by analyzing their telecommunication patterns. We apply k-means clustering analysis to describe contacts based on the frequency, direction, and duration of their phone calls and text messages with the focal participant. Identified “important contacts” satisfy the following criteria: they engage in i) mutual communication in both incoming and outgoing directions; ii) frequent communication in both incoming and outgoing directions, with the total number exceeding a certain threshold (i.e., 90% quantile); iii) at least one “long” consecutive text message exchange and one “long” call conversation, defined by exceeding certain thresholds (i.e., 90% quantile); and iv) communication that spans multiple days. An in-depth description of the k-means clustering approach to identify “important contacts” can be found in the supplementary material.

### Statistical analysis

We first provide descriptive statistics for the active and passive smartphone-derived measures of social activity and relevant demographic factors (gender, race, age, diagnosis). We then proceed to evaluate, for each patient, the correlation among active and passive social activity measures using an unsupervised clustering approach for multivariate time series by dynamic time warping distance [15], using the dtwclust R package [16]. The main advantage of dynamic time warping is the possibility to group time series according to their patterns or shapes even if these patterns are not synchronized. For the dynamic time warping distance calculation, we utilize the Euclidean norm metric, along with a symmetric local continuity constraint, with no windowing applied. We use a hierarchical clustering procedure in the primary analysis and the partitional clustering algorithm [17] in the secondary analysis to evaluate the sensitivity of results to the choice of clustering algorithm. We set the number of clusters in advance from two to five and we used the Calinski-Harabasz cluster validity index [18] to choose the optimal number of clusters (the higher the index the better the cluster size associated with it fits the data). We conduct cluster analyses first including mobile communication data for all contacts and then including communication data involving “important contacts” only. We evaluate the association between the social network features on primary outcomes (square-root of weekly count of outgoing calls, square-root of weekly count of outgoing texts, weekly average of self-reported digital social activity, weekly average of self-reported loneliness) using linear mixed models. All longitudinal regression models include a time factor (week), a seasonal factor, and the social network features. We call this the reduced model. In the full model, we include all other passive measures of mobile activity and self-reports of social activity. We fit reduced and full models first including mobile communication data on all contacts and then including only communication data involving “important contacts.” All tests of statistical significance were two-tailed. We considered 4 outcomes and for each outcome 6 social network predictors were tested considering both all contacts and important contacts, yielding 48 comparisons. Using a Bonferroni correction, we used an alpha = 0.05/48~0.001 to establish statistical significance. Statistical analysis was performed on R studio version 1.3.1073. All analyses were conducted under the complete case paradigm as in recent work of our group we showed that traditional single and multiple imputation approaches applied to intense longitudinal data, that are often found to have non-constant mean and variance over time (i.e., non-stationary), may introduce bias [19].

## Results

### Participant characteristics

**Fig. 1:**
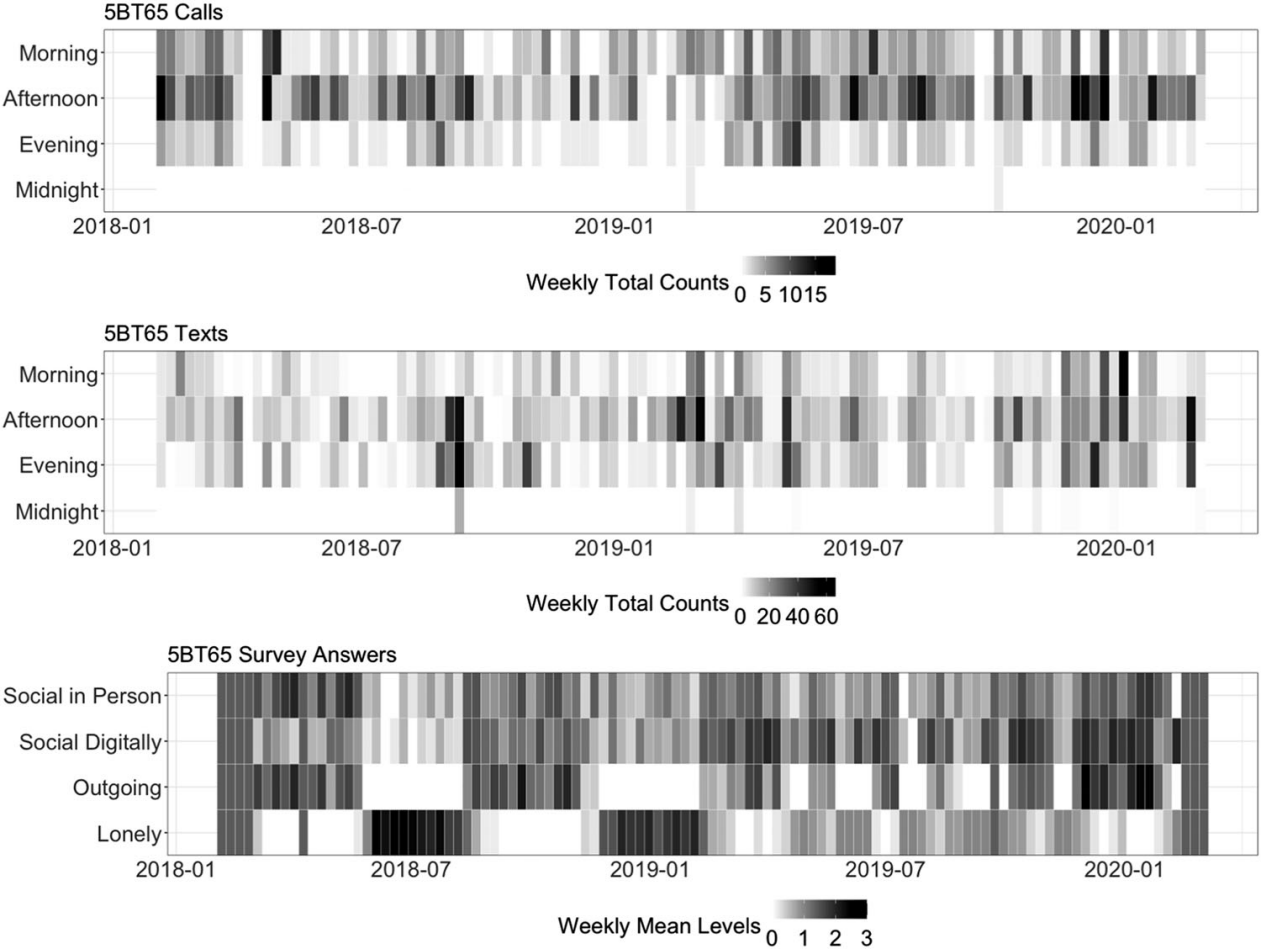
Weekly call/text counts and weekly mean levels of survey responses on social activity, loneliness, and outgoingness for a bipolar patient followed for two years. Specifically, levels of loneliness and outgoingness range from 0 (very slightly or not at all), 1 (a little), 2 (moderately), 3 (quite a bit), to 4 (extremely); levels of social interaction range from 0 (almost no interaction), 1 (little interaction more than superficial), 2 (meaningful interaction), 3(interaction with a close member) to 4 (an unusually deep conversation with another person).

The study included 9 Android user participants (4 females and 5 males aged 22 to 42) with follow-up spanning from 5 weeks to 208 weeks. Table S1 provides information on individuals’ diagnosis, years since diagnosis, and a list of medications during follow-up. Unique among mobile health studies in schizophrenia (SZ) and bipolar disorder (BD) patients, our sample includes information across 4244 person-days with 2538 complete observations. Four participants had a SZ diagnosis and five had a BD diagnosis. The average age was 34 and 32 for BD and SZ patients, respectively. Follow-up time was highly heterogeneous across subjects (Table 1) with weeks of follow-up spanning from 10 to 72 (mean = 30) for BD and from 5 to 208 (mean = 84) for SZ patients (p-value for differences = 0.33). Distribution of call and text features along with survey reports shows marked heterogeneity in mobile communication incoming and outgoing call and text activity across patients within each diagnostic group. Some patients displayed intense texting activity and little call activity and the opposite was found for other patients. Communication degree and reciprocity also differed substantially across subjects. Missing data on daily surveys spanned from 0% to 80%. There was no missing data in the sensor-based measures. Figure 1 illustrates the active and passive data streams for one participant. Weekly call and text counts by time of day are displayed in the top two figures and the bottom figure displays the weekly mean of social activity self-reported measures. During weeks when the patient reports high levels of loneliness, the patient reports low levels of social activity in person and digitally as well of feeling outgoing. During weeks when the patient reports high levels of loneliness, mobile and text activity appears reduced, particularly during the evening hours.

**Table 1:** Descriptive statistics of participant demographics, passive telecommunication data (text and call logs), EMA survey data, and survey missing rates.

	3UU85	4GS53	5CR39	5BT65	7NE49	8MJ89	9SU83	M3YZM	M8MXM
**DEMOGRAPHICS**									
Gender	Male	Male	Female	Male	Female	Female	Male	Male	Female
Age	27	24	64	42	37	26	23	22	34
Diagnosis	BP	SZ	BP	SZ	SZ	BP	BP	BP	SZ
Follow up (weeks)	23	15.57	25.29	108.57	208.71	20.86	72	9.71	5.43
**PASSIVE TELECOMMUNICATION DATA**									
Total number of call contacts	117	105	311	77	994	69	468	73	57
Total number of text contacts	59	73	90	29	189	1	178	77	47
Incoming call weekly counts	6.26 (6.09)	21.88 (10.54)	0.94 (1.28)	14.52 (11.15)	9.62 (7.31)	2.95 (2.56)	7.65 (3.59)	14.2 (14.59)	6.00 (1.55)
Outgoing call weekly counts	26.96 (26.23)	18.38 (9.47)	8.01 (5.19)	24.48 (20.36)	19.22 (12.98)	7.90 (7.09)	20.62 (10.21)	25.80 (36.79)	6.33 (4.84)
Received text weekly counts	72.91 (57.85)	305.00 (112.65)	14.42 (12.09)	40.04 (36.54)	41.24 (43.98)	0.00 (0.00)	42.68 (72.82)	148.9 (160.52)	410.17 (69.59)
Sent text weekly counts	186.22 (175.15)	353.56 (138.00)	13.91 (14.76)	96.24 (114.61)	61.12 (45.83)	0.14 (0.36)	136.94 (82.52)	127.7 (138.12)	407.17 (79.71)
Weekly call reciprocity	8.96 (10.28)	13.12 (6.22)	0.62 (1.15)	15.88 (14.2)	8.04 (6.67)	1.52 (1.91)	9.22 (5.31)	18.40 (22.53)	1.67 (1.37)
Weekly text reciprocity	84.52 (70.04)	360.38 (149.37)	13.34 (14.93)	40.68 (44.33)	44.40 (50.16)	0.00 (0.00)	49.31 (83.91)	174.6 (184.45)	396.67 (86.22)
Weekly number of incoming call contacts	4.26 (3.43)	7.94 (2.32)	0.80 (1.04)	5.40 (2.83)	4.93 (3.02)	2.19 (1.78)	5.57 (2.45)	5.10 (4.79)	5.50 (1.38)
Weekly number of outgoing call contacts	11.17 (6.83)	8.31 (2.98)	5.14 (3.24)	7.80 (4.72)	9.47 (6.11)	4.00 (3.41)	11.18 (4.89)	8.60 (9.61)	4.67 (1.86)
Weekly number of received text contacts	9.22 (3.77)	13.69 (3.61)	4.64 (2.42)	3.88 (2.07)	5.17 (5.08)	0.00 (0.00)	3.51 (5.72)	14.50 (12.7)	21.33 (2.16)
Weekly number of sent text contacts	10.04 (3.81)	8.81 (3.47)	2.68 (1.67)	4.00 (1.87)	4.18 (2.22)	0.14 (0.36)	13.39 (6.05)	15.30 (13.61)	14.50 (1.87)
**EMA SURVEY DATA**									
Weekly lonely	0.43 (0.39)	0.35 (0.38)	0.76 (0.76)	0.00 (0.00)	0.55 (0.51)	0.00 (0.00)	0.28 (0.54)	0.47 (0.45)	0.80 (0.62)
Weekly outgoing	1.95 (1.15)	1.49 (0.58)	0.81 (0.77)	1.78 (0.33)	1.90 (0.83)	1.50 (0.29)	1.80 (0.47)	3.13 (0.33)	0.73 (0.43)
Weekly social digitally	1.74 (0.8)	1.76 (0.45)	1.10 (0.56)	1.95 (0.94)	1.85 (0.51)	1.16 (0.64)	1.66 (0.47)	1.57 (0.6)	1.27 (0.27)
Weekly Social in person	1.92 (0.92)	1.66 (0.43)	1.00 (0.48)	2.05 (0.84)	2.80 (0.29)	2.32 (0.32)	1.62 (0.48)	3.07 (0.15)	1.90 (0.54)
**MISSING DATA**									
Days with complete survey data	99 (61.11%)	29 (26.36%)	127 (71.35%)	544 (71.48%)	958 (65.53%)	53 (36.05%)	98 (19.41%)	18 (26.09%)	39 (100%)

EMA SURVEY DATA summarizes daily survey responses on levels of loneliness and feeling outgoing, ranging from 0 (very slightly or not at all), 1 (a little), 2 (moderately), 3 (quite a bit), to 4 (extremely). Similarly, weekly reports on social interaction summarize daily self-evaluated social interaction levels, ranging from 1 (almost no interaction), 2 (little interaction more than superficial), 3 (meaningful interaction), 4 (unusually deep interaction), covering in-person and digital interactions.

### Identification of important contacts

Adopting the k-means clustering approach, we were able to identify contacts who entertained sustained and intense communication with the participant. Cluster analysis of texts was unnecessary for a participant with only one text contact. One participant did not report the number of people they rely on for emotional support during clinical interviews. Table S2 in the supplementary material lists the call and text features used for k-means clustering for important contact identification. Figures S1–S9 visualize the clusters we identified for each participant and Table S3 summarizes the results. Identified important contacts via phone calls and text messages often overlapped. The number of important contacts for calls ranged from 2 to 9 and for texts ranged from 1 to 8 across subjects. Important contacts identified via interviews give the range of number of people that subjects report they rely on for emotional support every month over in-person visits. The number of important contacts identified via the k-means clustering approach was included in the range reported during clinical interviews.

### Cluster analysis

**Fig. 2:**
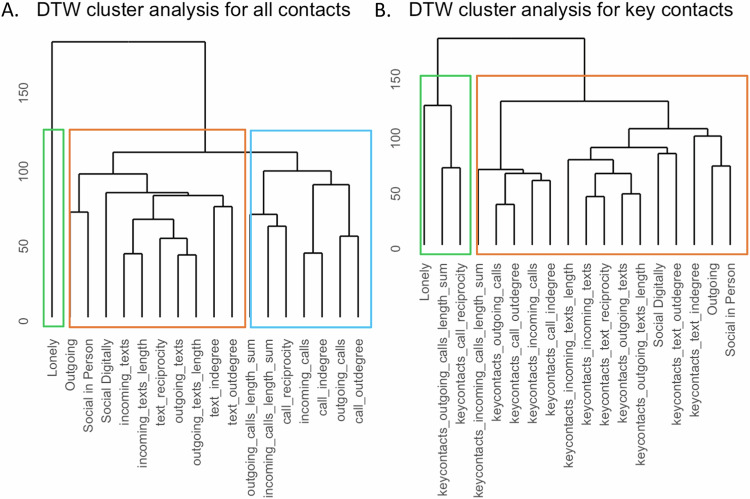
Cluster analysis result for one bipolar patient. **A** The hierarchical and partitional clustering analysis agree with the choice of three clusters. Loneliness clusters separately, and social network degree clusters with self-report of social activity. Social network reciprocity measures clusters with call and text duration. **B** When considering mobile communication data involving only “important contacts” the hierarchical and partitional clustering analysis agrees in the choice of 2 clusters. Loneliness clusters with mobile communication features, separately from self-report of social activity.

For each participant, we explored patterns across the multivariate time series of active and passive measures of social activity via hierarchical clustering by dynamic time-warping distance. The analysis shows the correlation among the features with tighter trees, indicative of a stronger correlation when only mobile communication data involving important contacts is used for the passive measures. The number of clusters detected using the CH index is lower and the tree branches are shorter when only mobile communication data involving important contacts is used compared to when the whole mobile communication data is included. This is an indication that when important contact information is used, passive data better reflects self-report of social activity and/or loneliness. Time series of active and passive data cluster differently across patients, reflecting heterogeneity in time series dynamics and their correlation among subjects. For one BD subject in Fig. 2 who was followed for 100 weeks (5BT65) the clustering analysis identified three clusters. Loneliness clustered separately, the social network feature of mobile communication degree clustered with self-report of social activity, and the social network feature of reciprocity clustered with call and text duration. When considering mobile communication data involving only “important contacts” the clustering analysis identified two clusters. Loneliness clustered with mobile communication features of outgoing call length and reciprocity, separately from self-report of social activity and other mobile communication features. For another BD subject followed for 25 weeks (5CR39, Figure S10) the clustering analysis identified two clusters and self-reported measures of social activity clustered separately from mobile communication features. When considering mobile communication data involving only “important contacts” two clusters were identified with self-reported measures of social activity now clustering with a degree of mobile communication. Figure S11 displays results for a participant with SZ diagnosis (M8MXM) for whom the clustering analysis identified three clusters. Loneliness clustered with other self-report measures of digital and in-person social activity separately from other passive data measures of mobile communication. When considering mobile communication data involving only “important contacts” the clustering analysis identified two clusters. Loneliness clustered with mobile communication features of network degree in addition to self-report of social activity, separately from other features of mobile communication. Some similar patterns arise across subjects. The social network feature of mobile communication degree clusters more closely with social activity self-report and loneliness than other mobile communication data, when including only important contacts.

### Regression analyses

**Fig. 3:**
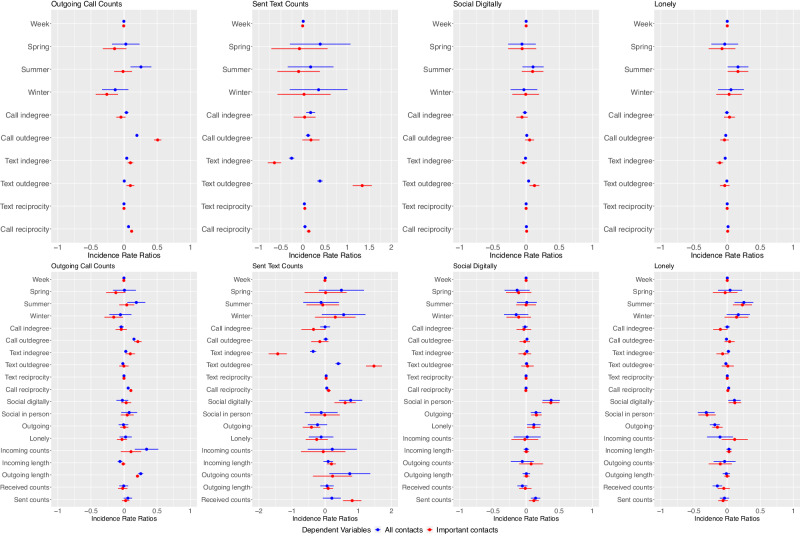
Linear mixed effect model analyses results. The top panels present results from the reduced model that includes seasonality and social network-derived features of mobile communication as predictors. The bottom panels present results from the full model which in addition to the seasonality and social network-derived features includes other more common passive features of mobile communication. Each panel shows estimates from the model fitting for each outcome considered (Outgoing calls and text counts, self-reported in-person and digital social activity). Estimates in blue indicate a model that includes all data and estimates in red indicate a model that includes passive data for important contacts only.

We investigated the association of social network features of mobile communication (outgoing call degree, incoming call degree, outgoing text degree, incoming text degree, call reciprocity, text reciprocity) with participant willingness to engage in mobile communication (outgoing call and text counts), patient’s perception of digital activity, and loneliness perception using linear mixed models. We quantified such associations considering all call and text data as well as including only call and text data involving important contacts. Figure 3 presents the results across outcomes of interest for a reduced model (top). The reduced model includes seasonality terms and social network features. The full model (bottom) includes seasonality terms, social network features, as well as call and text features, and self-reported social activity measures (in-person social activity, digital social activity, feeling outgoing, loneliness). Results are shown for all mobile communication data (blue) and including only key contacts mobile communication data (red). The analyses underscore that, among the social network features, the mobile communication network degree measure is more strongly associated to the outcomes than the reciprocity measure. Comparing reduced to full models, social network features of mobile communication degree remain independently associated with the outcomes even after accounting for other mobile communication features, except for self-report of digital social activity for which we find that, among mobile communication features, count of sent texts is the strongest predictor. Comparing models including all mobile communication data (blue) and only important contacts mobile communication data (red), the regression analyses confirm the cluster analyses results in that associations of social network features with the outcomes are stronger and more significant in the models including only important contacts mobile communication data.

In the full models including only key contacts outgoing call degree was independently and positively associated with outgoing count of calls (beta = 0.5, 95% confidence interval = [0.45,0.56], *p*-value < 0.0001). Furthermore, incoming text degree was positively associated with the count of outgoing calls (beta = 0.11, [0.07,0.15], *p*-value < 0.0001). Outgoing text degree was positively associated with outgoing counts of texts (beta = 1.53, [1.28,1.78], *p*-value < 0.0001). Finally, we found suggestive evidence of a negative association of incoming call (IC) and incoming text (IT) degree with loneliness, albeit the significance did not meet the multiple comparison threshold (beta_IC_ = −0.11, [−0.21, −0.002], *p*-value_IC_ = 0.04, beta_IT_ = −0.07, [−0.18,0.02], *p*-value_IT_ = 0.12). Among the mobile communication features, incoming call and incoming text degree were the ones found most closely associated with loneliness. In-person social activity was the strongest predictor of loneliness (beta = −0.3, [−0.43, −0.17], *p*-value < 0.0001).

## Discussion

We explored the usefulness of smartphone mobile communication data and social network features derived from it as indicators of self-reported perception of social activity and perception of loneliness in people with SZ and BD disorder. Findings suggested these data show promise as correlates of self-reported measures of social activity and loneliness. Capturing mobile communication features for important contacts on mobile communication degree appeared more promising predictors. Our results indicate that in this sample, individuals with SZ and BD may have a smaller number of important contacts who play a more meaningful role in their social network rather than a larger number of distant contacts. This was underscored by two separate types of analyses. A cluster analysis showed a tighter relationship among self-reported measures of social activity and loneliness with mobile communication degree involving important contacts. A generalized linear model analysis identified marginally significant associations of mobile communication degree involving “important contacts” with loneliness.

We also investigated whether social network features of mobile communication are independently associated with individuals’ willingness to engage in mobile communication, captured by the outgoing count of calls and texts. Indeed, our analyses underscore a strong association between social network degree and these outcomes. Again, this association is found stronger when “important contact” data only is included. Finally, among the mobile communication features, the count of outgoing text was the only one found to be independently associated with self-report of digital social activity.

This study adds to the rapidly growing body of work capitalizing on the ubiquity of smartphones for capturing naturalistic data in people with BD disorder and SZ. In the current study, we focused on social behavior and loneliness perception, with the goal of guiding best practices in extracting relevant features of mobile communication data that can capture social behavior. There is interest in considering multiple domains of passive data that can capture social activity/isolation and loneliness [6]. In another article, we consider mobility features (derived from passive data) as well [20]. In this study, we consider only mobile communication data to reduce issues of multiple testing due to sample size considerations.

We find that filtering mobile communication data with the identification of communication of “important contacts” helps to reduce noise in the data. We further provide preliminary evidence that social network features of mobile communication are independent correlates of participants’ engagement in mobile communication activity, perception of social activity, and loneliness.

Our study contributes to the existing body of literature that highlights the potential of utilizing smartphone technology to detect and measure social activity. While previous studies in individuals with SZ and BD disorder have primarily focused on alternative digital data, such as GPS, voice recordings, frequency, and duration of mobile communication to passively capture social behavior [5, 21], our research expands upon this knowledge by presenting preliminary evidence on the feasibility and relevance of extracting social network features from mobile communication logs in this specific patient population. Communication logs, from which all call and text events are extracted, are a form of passive data as they require no involvement of and impose no burden on the participant. We provide preliminary evidence in this population that adopting a social-network perspective in the processing of mobile communication data can allow to capture the quality of mobile communication in addition to the quantification of such activity [8, 10]. Furthermore, we offer initial insights into the feasibility and significance of identifying “important contacts” for enhancing the accuracy and meaningfulness of communication network features in capturing social activity and predicting perceptions of loneliness. It is of interest, in the future with a larger sample size, to evaluate the clinical and real-world implications of social network size in the SZ and BD population, leveraging the passively derived features we here evaluated to reduce patients’ burden.

Our study has several strengths. First, the multi-year follow-up period. Following BD and SZ patients over an extended period allows for the examination of changes and patterns in social activity and loneliness more effectively. Large variability in follow-up time is very common in digital studies, especially in studies that make use of participant’s own devices (which ultimately will make the approach scalable). A strength of our study is that we use all the available data rather than selecting only the overlapping data, as many other studies do. Second, the study leverages mobile technologies, specifically smartphones, to construct novel markers of social activity based on passive data on patients’ social activity and validate them against self-reported active data. By using smartphones, we were able to capture real-time information about social behavior in a non-intrusive manner, providing valuable insights into patients’ daily lives. Third, our study recognizes the importance of a social network perspective in understanding mental illness and loneliness. By incorporating social network features derived from mobile communication data, we were able to explore the role of mobile social connections in patients’ perception of engagement in social activity and feelings of loneliness.

Some limitations in our study should also be noted. Although passive-sensing and digital phenotyping are promising avenues for psychological science to study people unobtrusively in real life [22, 23], their application still warrants more systematic validation studies (e.g., [24]), data processing guidelines [25], and tailored analysis methods [26]. A limitation of our empirical demonstration is that we examined a small sample, limiting generalizability. To address this shortcoming, in addition to a traditional linear mixed effect model analysis we implement a cluster analysis considering an n-of-1 approach. This approach further accommodates large patient heterogeneity in diagnosis and follow-up time. The validity of the cluster analysis relies of the number of observations for each participant rather than the sample size. The long follow-up of the Bipolar Longitudinal study (also referred to as the “one-hundred-person-year study”) is a key strength of our work.

In the current study, we processed the passive and active smartphone data at the weekly level. Different days of the week typically have their own phone communication patterns as we have shown [27]. The use of weekly averages obviates the need to model day-level trends and mitigates issues of missing data in mHealth, which is generally ignored. Furthermore, our group has shown that complete case daily data analyses or traditional multiple imputations can introduce bias [19]. Future studies should examine the interplay between mobile communication features and self-reports of social activity and loneliness in larger and more diverse populations capturing data at the daily level with appropriate approaches that account for potential missing data not at random and non-stationarity [28].

This study contributes to our understanding of social interaction among individuals with SZ and BD disorder by leveraging smartphone sensors to examine fine-grained mobile communication, and mobile communication network features in particular, as a proxy for social activity and loneliness perceptions in daily life settings. By extracting and summarizing rich sensor data from individuals’ smartphones, this research demonstrates the ability to identify key features that strongly correlate with individuals’ perceptions of social activity and loneliness. Through this approach, the study offers valuable insights into the complex dynamics of social life in these populations, shedding light on the potential of smartphone data as a valuable resource for monitoring and potentially intervening in social interactions and investigating their relationships with loneliness perceptions.

## Supplementary information

Supplemental Material

## Data Availability

The data that support the findings of this study are available from the corresponding author, LV, upon reasonable request.
