# Supplementary material for: Smartphone-based markers of social connectivity in schizophrenia and bipolar disorder
Source: NPP Digit Psychiatry Neurosci. 2024 Jul 24;2:12. doi: 10.1038/s44277-024-00013-w (PMC12624914; doi:10.1038/s44277-024-00013-w)
Supplement: Supplementary file 1 — Supplemental Material [file 44277_2024_13_MOESM1_ESM.pdf]

Smartphone-based markers of social networks in schizophrenia and bipolar disorder

Linda Valeri, Xiaoxuan Cai, Aijin Wang, Habiballah Rahimi Eichi,
Einat Liebenthal, Scott Rauch, Dost Ongur, Russell Schutt, Lisa Dixon,
*Jukka-Pekka Onnela, *Justin Baker, (* denotes co-last authors)

Important contacts identification via K-means clustering

Supportive relationships are essential for coping with stress and mental illness. Particularly, having a close, mutually confiding relationship might be critical to mitigate and improve psychiatric symptoms. Typically, supportive roles are filled by close relatives, friends, or agency professionals. Contacts identified via calls and texts are composed of random contacts (e.g., scam calls, food delivery calls, and medical appointment reminders) and important or key contacts, who provide subjects with sustained emotional (e.g., reflecting concern, providing intimacy, listening), instrumental (e.g., providing material aid), or informative (e.g., problem-solving suggestions) support. Exchanges with random contacts are typically brief, unidirectional, and infrequent. In contrast, exchanges with important (key) contacts are typically bi-directional, of varying length (including both lengthy and brief exchanges) and occur over multiple days and a wide range of time.

We used k-means clustering on anonymized summary features of calls and texts in conjunction with relevant in-clinic interview questions to identify important contacts for each participant. Important contacts identified via phone calls and text messages may overlap. Please refer to Table S1 below for texts and phone calls features used to derive clusters identifying important contacts. Note that in determining the optimal number of clusters for K-means analysis, we did not follow the popular “elbow curve method”, which identifies the point at which the average distance from the centroid falls abruptly. The purpose of the “elbow curve method” is to stop increasing the number of clusters when adding a new cluster does not significantly improve the data modeling. In contrast, the clustering objective in our study is to identify a small group of important contacts of the patients. As a result, we cluster contacts to the finest degree and then select the cluster(s) that satisfy all predetermined criteria and are therefore deemed to include data on important contacts. Cluster analyses of most participants result in the selection of a single cluster that meets all criteria for important contacts. If the number of important contacts identified by multiple clusters matches the number of important contacts reported in the clinic interview, then only the cluster with the highest level of communication is selected for important contacts. Table S2 shows the optimal number of clusters chosen via k-means analysis of phone calls and text messages, as well as their identified number of important contacts, compared to the range of monthly reported number of people on whom participants rely for emotional support.

Supplementary Tables

**Subject**	**ER visits**	**Hospitalizations During Study**	**Diagnosis**	**Years since Diagnosis**	**Medications***
4GS53	0	0	SZA-BP[SCID]	3	aripiprazole 5 mg<br>divalproex 250 mg
5CR39	0	0	SZ[SCID]	15	benztropine 1 mg<br>haldol 20 mg<br>zolpidem 12.5 mg
5BT65	1	0	BP1[SCID]	41	bupropion 75 mg<br>clozapine 25 mg<br>lamotrigine 100 mg<br>vortioxetine 20 mg
7NE49	0	0	SZA-BP[SCID]	>10	carbamazepine 200 mg<br>clomipramine 75 mg<br>diazepam 5 mg<br>melatonin 2.5 mg<br>perphenazine 2 mg<br>propranolol 10 mg
8MJ89	Unknown	Unknown	BP1[SR]		None
9SU83	7	5	BP1[SCID]	6	divalproex 500 mg<br>lithium 300 mg
M3YZM	2	0	BP1[EHR]		None
M8MXM	0	0	MDD [EHR]		bupropion 150 mg

Table S1: Information on diagnosis, years since diagnosis, hospitalizations, and list of medications during follow-up for BLS cohort participants included in the study. LEGEND: SCID, Structured Clinical Interview for DSM; SR, Self-reported; EHR, electronic health record. Note: Medications and dosages were changing throughout the study.

Call features	Text features
number of incoming calls	number of incoming texts
total length of incoming calls	total length of incoming texts
number of outgoing calls	number of outgoing texts
total length of outgoing calls	total length of outgoing texts
number of long calls	number of excessive text exchanges
number of days of contact	number of days of contact

Table S2: Anonymized call and text features used for k-means clustering for important contacts identification.

Participant	Number of clusters		Number of important contacts		
	Call	Text	Call	Text	Interview
3UU85	12	10	6	2	5+
4GS53	12	12	5	4	3-4
5CR39	12	10	5	1	1-3
5BT65	12	10	5	4	5-7
7NE49	12	10	7	4	4-7
8MJ89	12	-	3	1	4-5
9SU83	12	10	9	8	0-8
M3YZM	12	10	7	3	NA
M8MXM	12	10	2	5	9-10

Table S3: Number of clusters for k-means analysis and number of important contacts identified via phone and text features, respectively. Important contacts are identified leveraging the entire follow-up; identified important contacts via call and text data can overlap. Number of people that individuals rely on for emotional support was also obtained via monthly in-person interviews and we here show the range of reported number. Cluster analysis for text is unnecessary for participant 8MJ89, who had only one text contact.

Supplementary Figures

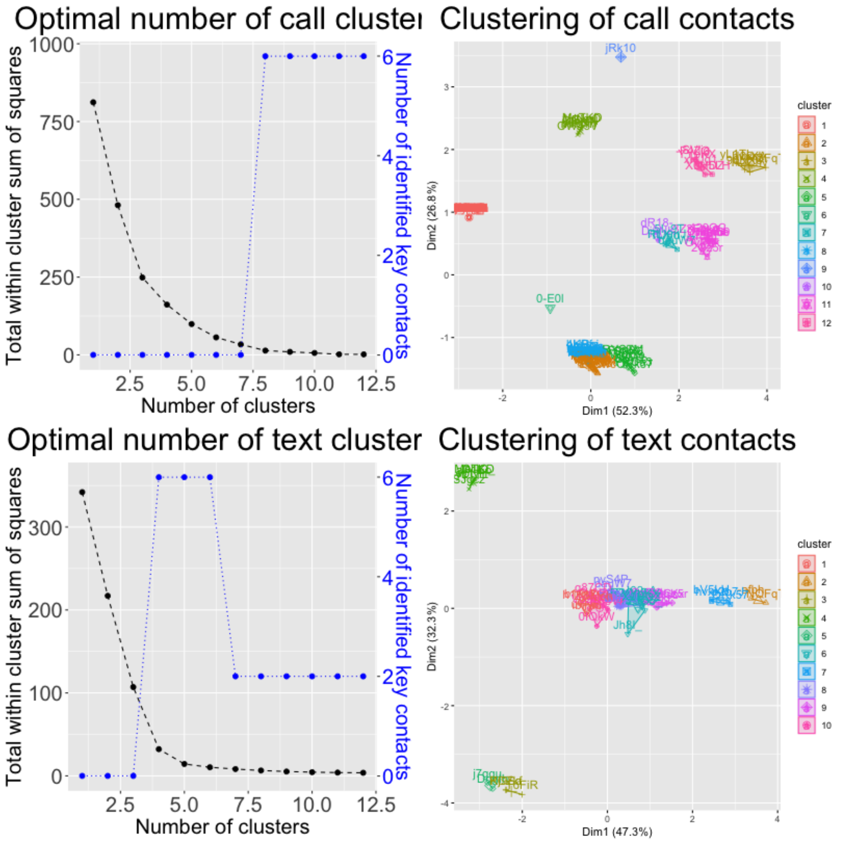

Figure S1: K-means clustering analysis of important contacts identification via call features (top panel) and text features (bottom panel) for participant 3UU85. The left panel illustrates the robustness of the number of clusters in the the number of identified important contacts for calls and texts, respectively. The right panel depicts the clustering of contacts based on the number of clusters selected for calls and texts, respectively.

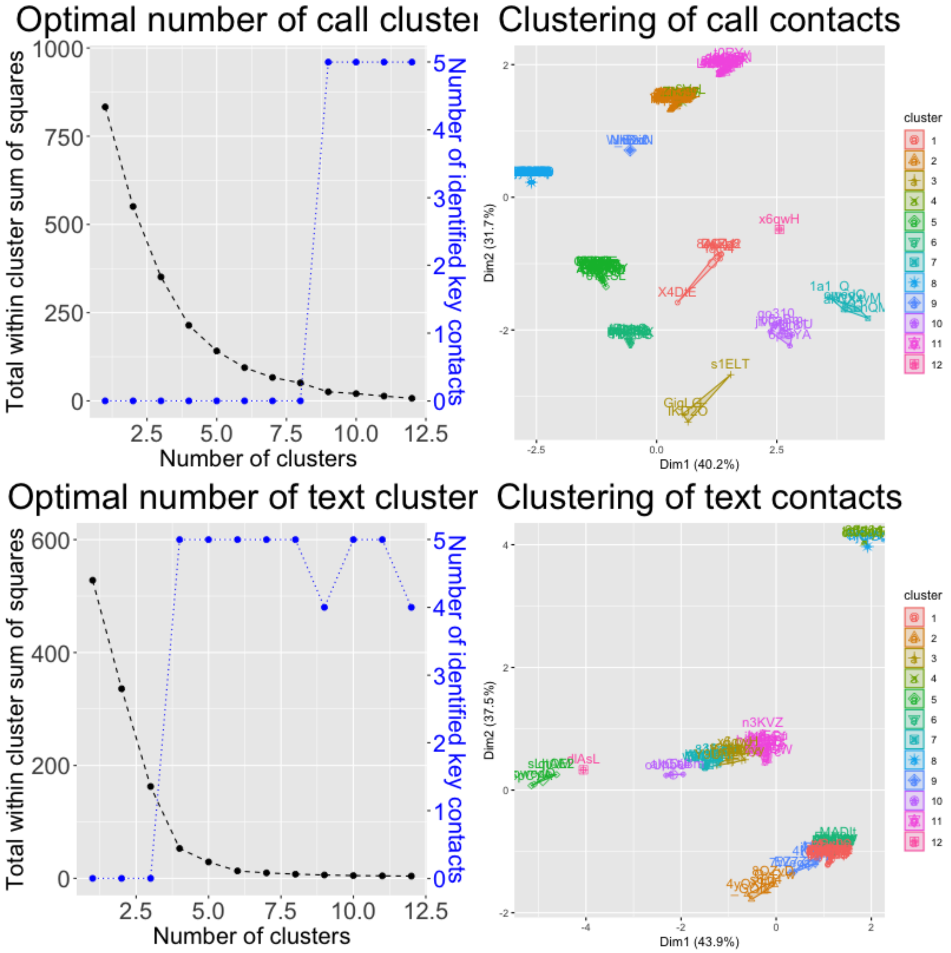

Figure S2: K-means clustering analysis of important contacts identification via call features (top panel) and text features (bottom panel) for participant 4GS53. The left panel illustrates the robustness of the number of clusters in the the number of identified important contacts for calls and texts, respectively. The right panel depicts the clustering of contacts based on the number of clusters selected for calls and texts, respectively.

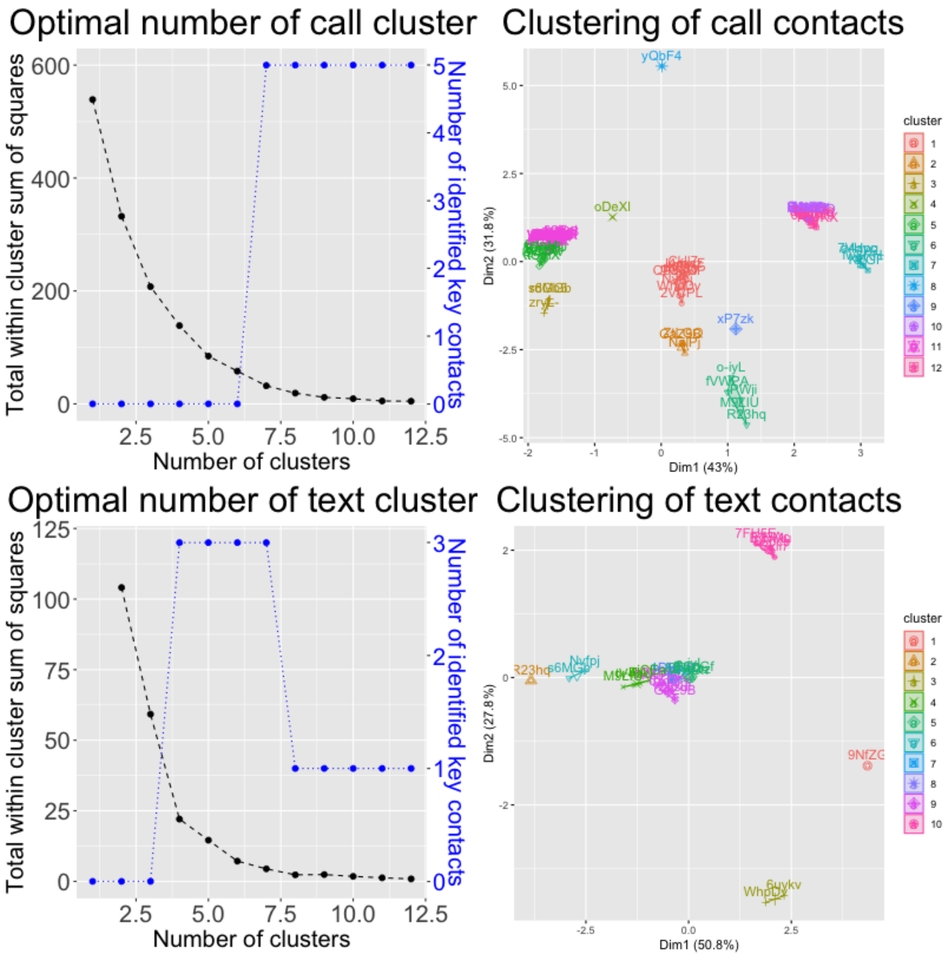

Figure S3: K-means clustering analysis of important contacts identification via call features (top panel) and text features (bottom panel) for participant 5CR39. The left panel illustrates the robustness of the number of clusters in the the number of identified important contacts for calls and texts, respectively. The right panel depicts the clustering of contacts based on the number of clusters selected for calls and texts, respectively.

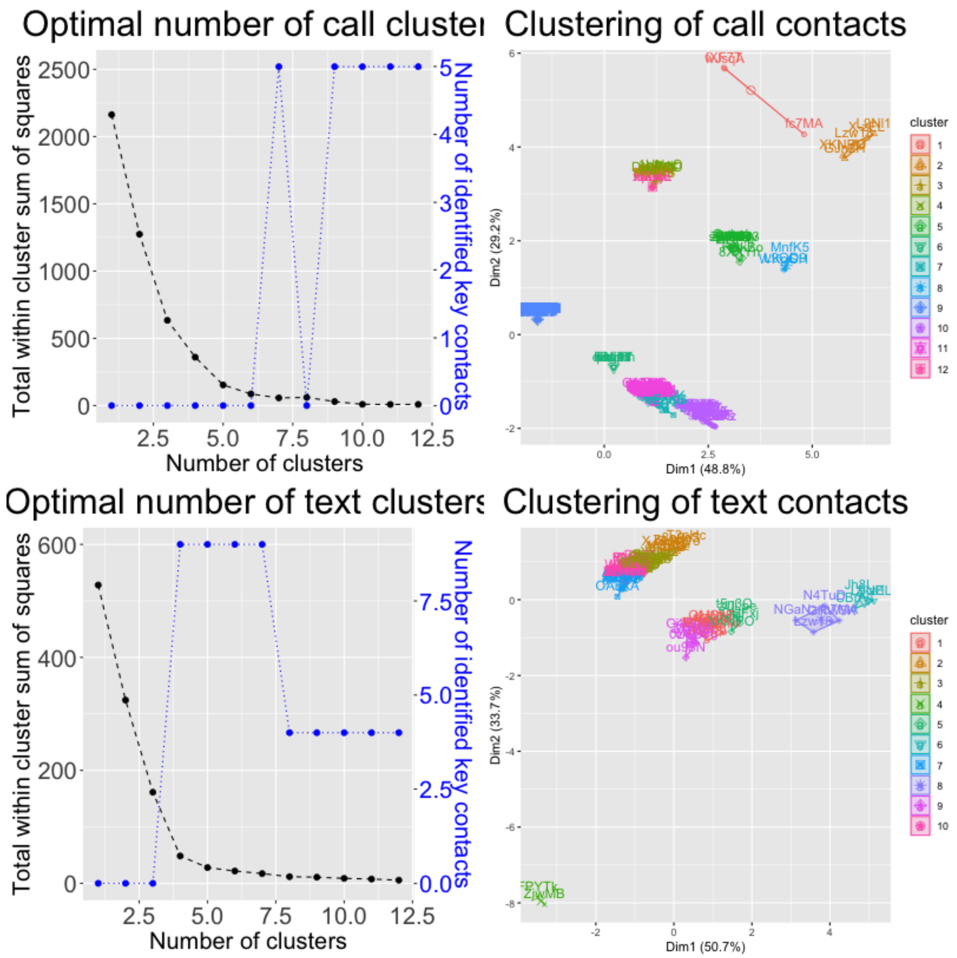

Figure S4: K-means clustering analysis of important contacts identification via call features (top panel) and text features (bottom panel) for participant 5BT65. The left panel illustrates the robustness of the number of clusters in the the number of identified important contacts for calls and texts, respectively. The right panel depicts the clustering of contacts based on the number of clusters selected for calls and texts, respectively.

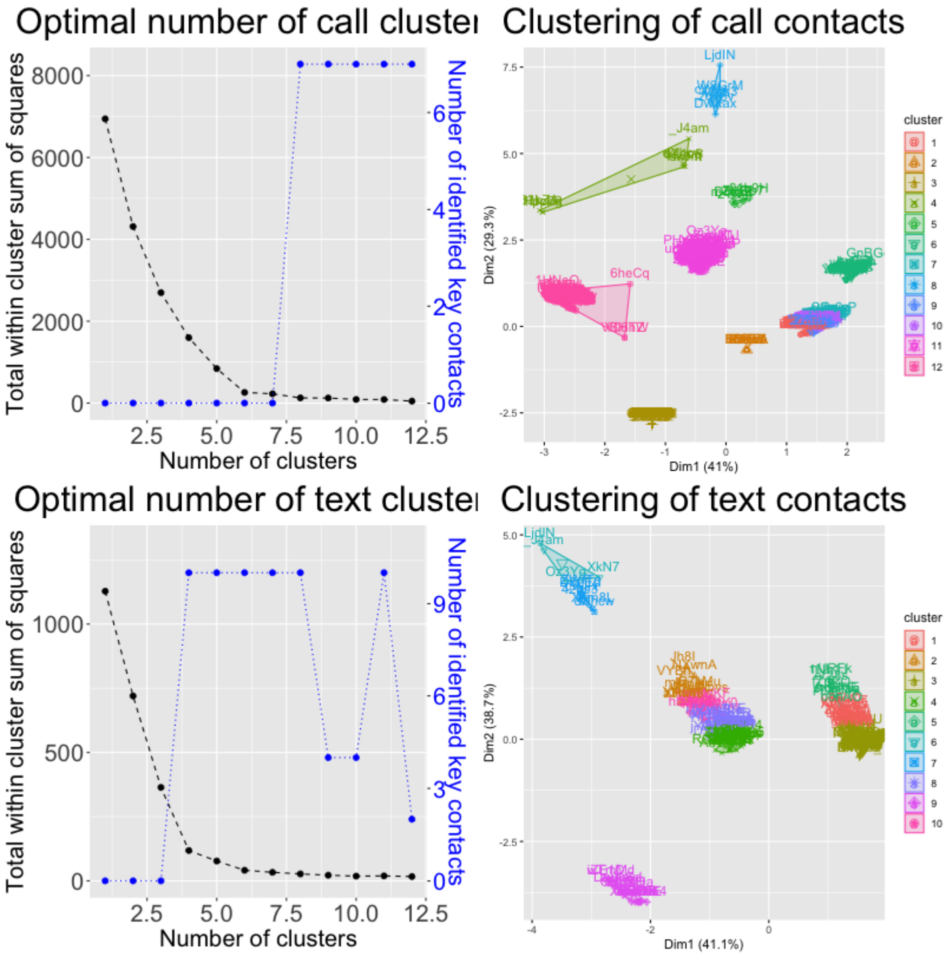

Figure S5: K-means clustering analysis of important contacts identification via call features (top panel) and text features (bottom panel) for participant 7NE49. The left panel illustrates the robustness of the number of clusters in the the number of identified important contacts for calls and texts, respectively. The right panel depicts the clustering of contacts based on the number of clusters selected for calls and texts, respectively.

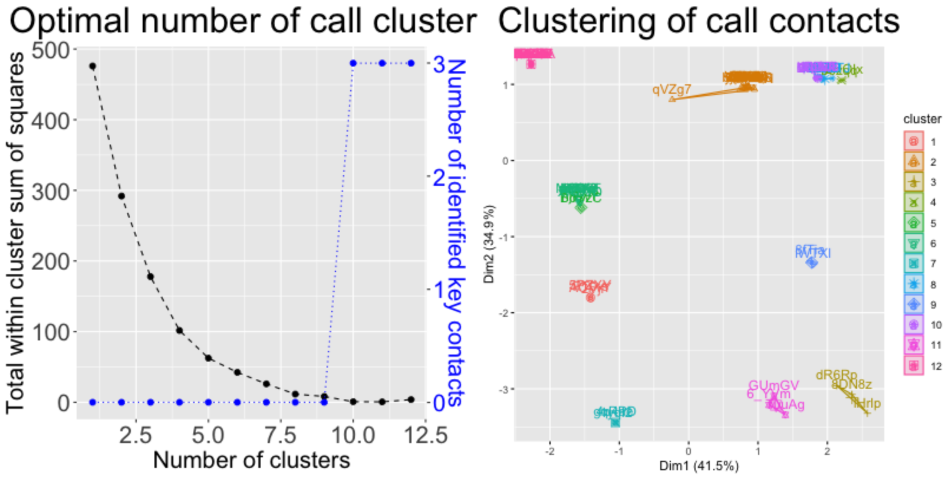

Figure S6: K-means clustering analysis of important contacts identification via call features for participant 8MJ89. The left graph illustrates the robustness of the number of clusters in the the number of identified important contacts for calls. The right graph depicts the clustering of contacts based on the number of clusters selected for calls.

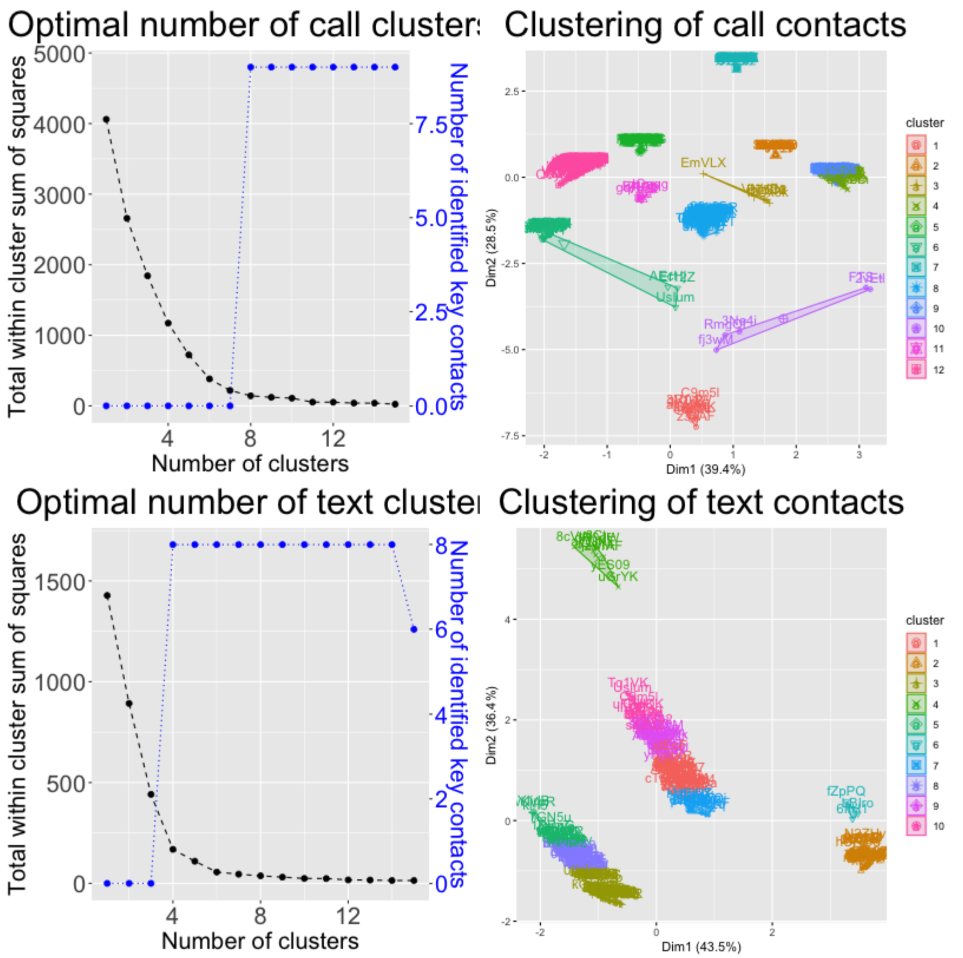

Figure S7: K-means clustering analysis of important contacts identification via call features (top panel) and text features (bottom panel) for participant 9SU83. The left panel illustrates the robustness of the number of clusters in the the number of identified important contacts for calls and texts, respectively. The right panel depicts the clustering of contacts based on the number of clusters selected for calls and texts, respectively.

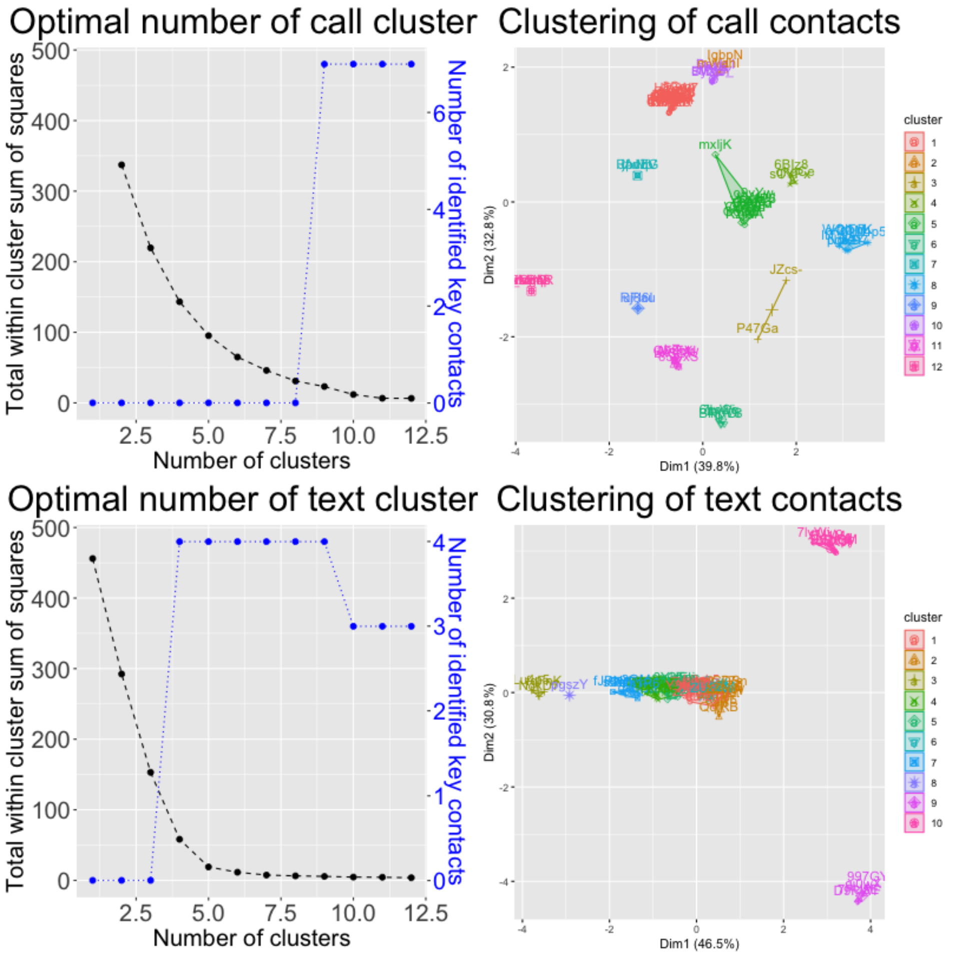

Figure S8: K-means clustering analysis of important contacts identification via call features (top panel) and text features (bottom panel) for participant M3YZM. The left panel illustrates the robustness of the number of clusters in the the number of identified important contacts for calls and texts, respectively. The right panel depicts the clustering of contacts based on the number of clusters selected for calls and texts, respectively.

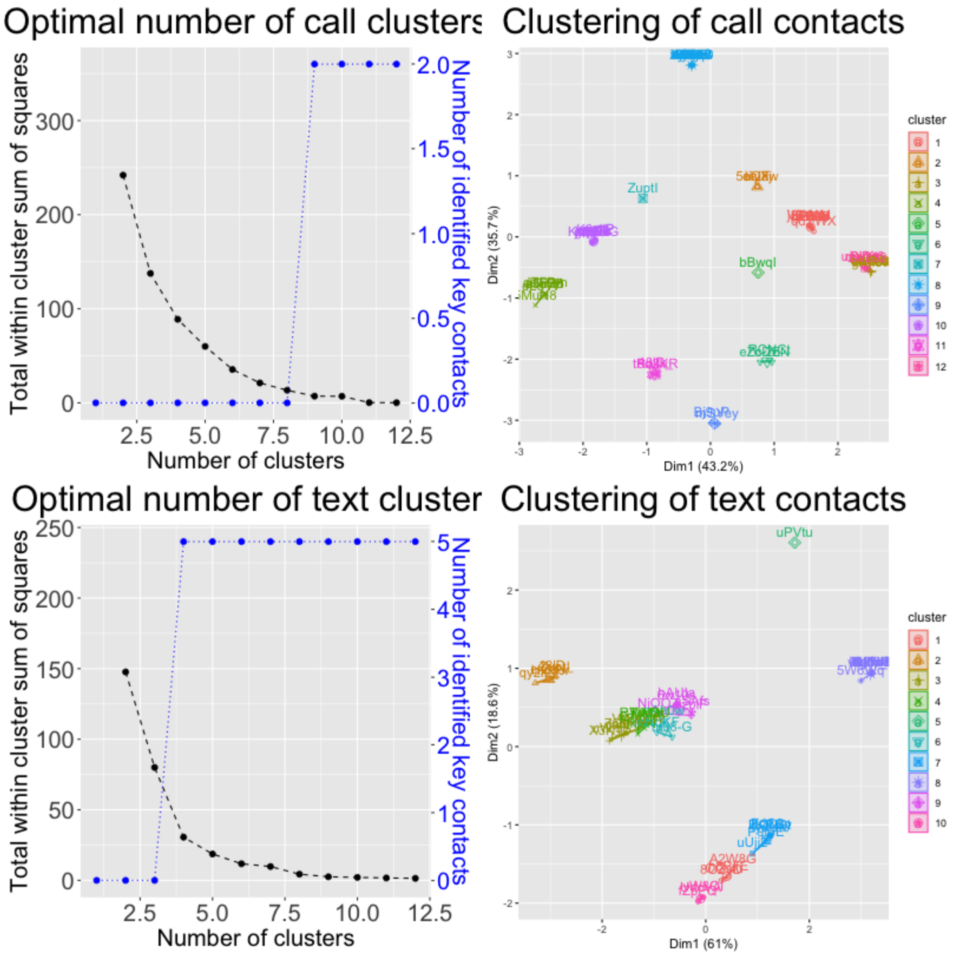

Figure S9: K-means clustering analysis of important contacts identification via call features (top panel) and text features (bottom panel) for participant M8MXM. The left panel illustrates the robustness of the number of clusters in the the number of identified important contacts for calls and texts, respectively. The right panel depicts the clustering of contacts based on the number of clusters selected for calls and texts, respectively.

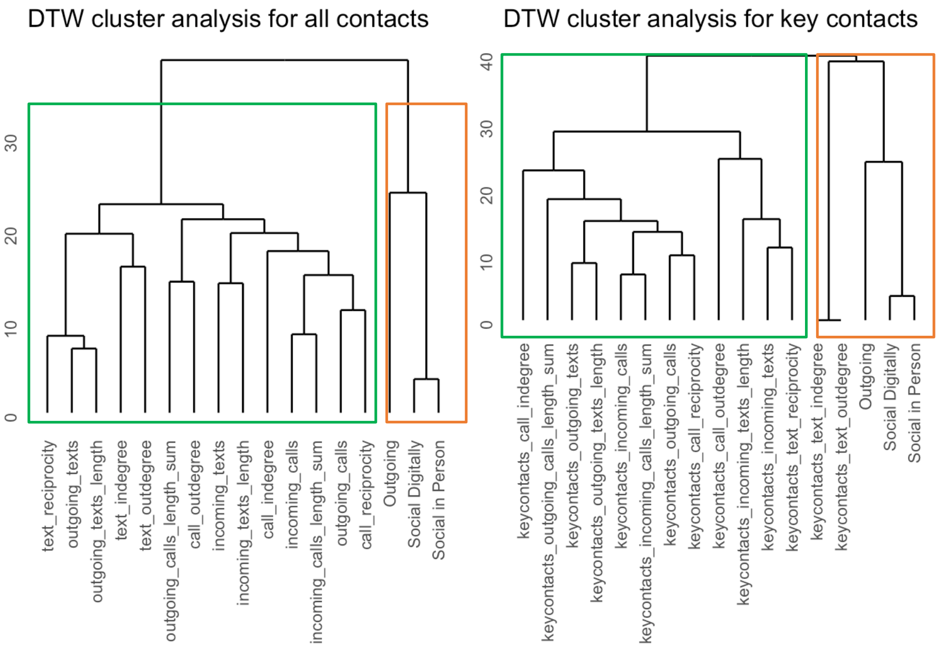

Figure S10: Cluster analysis result for one bipolar patient. (A) The hierarchical and partitional clustering analysis agree with the choice of 2 clusters. Social activity self-report measures cluster separately, social network degree clusters with other passive measures of social activity. (B) When considering mobile communication data involving only "important contacts" the hierarchical and partitional clustering analysis agree in the choice of 2 clusters. Social activity self-report measures cluster with social network degree, separately from other passive measures of social activity.

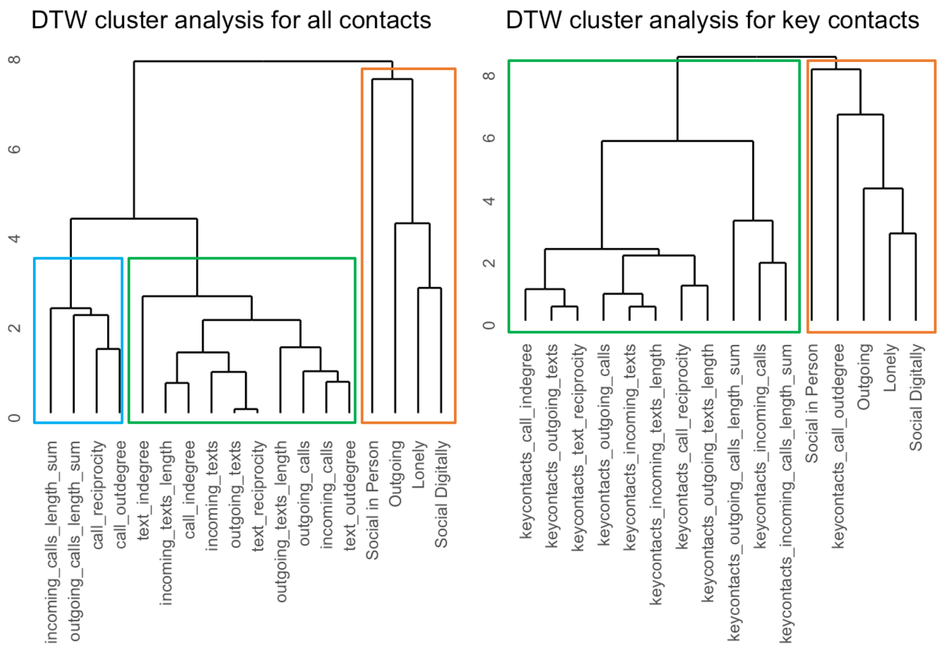

Figure S11: Cluster analysis result for one schizophrenia patient. (A) The hierarchical and partitional clustering analysis agree with the choice of 3 clusters. Social activity and loneliness self-report measures cluster separately, social network degree clusters with other passive measures of social activity. (B) When considering mobile communication data involving only "important contacts" the hierarchical and partitional clustering analysis agree in the choice of 2 clusters. Social activity and loneliness self-report measures cluster with social network degree, separately from other passive measures of social activity.
